# Curcumin and Cancer Stem Cells: Epigenetic Mechanisms Underlying Therapeutic Resistance and Tumor Relapse

**DOI:** 10.3390/ijms27156945

**Published:** 2026-08-02

**Authors:** Juie Nahushkumar Rana, Jayashri Ghosh, Sohail Mumtaz

**Affiliations:** 1 Fels Cancer Institute for Personalized Medicine, Lewis Katz School of Medicine, Temple University, Philadelphia, PA 19140, USA; tuu67213@temple.edu; 2 Department of Chemical and Biological Engineering, Gachon University, 1342 Seongnam-daero, Sujeong-gu, Seongnam-si 13120, Republic of Korea

**Keywords:** curcumin, cancer stem cells, epigenetic plasticity, DNA methylation, histone remodeling, non-coding RNA, therapeutic resistance, tumor relapse

## Abstract

Cancer stem cells (CSCs) drive therapeutic resistance, metastasis, and tumor recurrence through reversible transitions among stem-like, differentiated, epithelial, and mesenchymal states, which are sustained by interconnected epigenetic mechanisms. To our knowledge, this is the first review to integrate curcumin-mediated regulation of DNA methylation, chromatin remodeling, and non-coding RNAs within a single CSC plasticity framework and to propose the concept of an “epigenetic collapse of CSC plasticity” as a mechanistic explanation for how curcumin may weaken stemness, state switching, and adaptive treatment resistance. Evidence was critically evaluated through structured searches of PubMed/MEDLINE, Scopus, Web of Science Core Collection, Google Scholar, and citation tracking, while direct curcumin–epigenetic evidence was distinguished from independent CSC evidence and inferential mechanistic links. Curcumin has been reported to modulate DNMT1 and locus-specific DNA methylation; regulate HDACs, p300/CBP, EZH2, H3K27me3, and BMI1; and alter selected microRNA, long non-coding RNA, and circular RNA pathways, with comparatively stronger evidence involving the miR-34 family, miR-200c, miR-21, H19, and circHN1. However, current evidence is constrained by the predominance of bulk cancer-cell models, heterogeneous formulations and exposure conditions, and the scarcity of epigenetic rescue experiments combined with rigorous functional CSC assays. By unifying previously fragmented epigenetic evidence, this review advances a new evidence-weighted model in which curcumin may suppress CSC persistence not through a single molecular target, but by destabilizing the multilayer epigenetic circuitry that enables plasticity. Curcumin should therefore be regarded as a context-dependent, multilayer epigenetic modulator rather than an established CSC-eradicating therapy, and its translational relevance requires validation in prospectively defined CSC models with pharmacologically justified delivery and exposure conditions.

## 1. Introduction

Despite major advances in precision oncology, immunotherapy, and targeted therapeutics, metastatic progression, tumor recurrence, and acquired treatment resistance remain major causes of therapeutic failure and cancer-related mortality [1,2,3]. A growing body of evidence implicates cancer stem cells (CSCs), a functionally distinct and highly tumorigenic subpopulation of malignant cells, in these clinical outcomes [2,3]. CSCs are characterized by their capacity for self-renewal, tumor propagation, and differentiation into heterogeneous cancer cell lineages, although these properties are not necessarily confined to a permanently fixed cell population [2]. Compared with more differentiated tumor cells, CSC-enriched populations can display enhanced DNA-damage repair, quiescence, drug-efflux capacity, metabolic adaptability, resistance to apoptosis, and phenotypic plasticity, which together increase their tolerance of chemotherapy and radiotherapy [2,3]. CSCs that persist after treatment may consequently contribute to tumor repopulation, metastatic dissemination, and disease relapse [2,3]. Consistent with these functions, enrichment of CSC-associated phenotypes or markers has frequently been associated with aggressive disease, treatment resistance, and unfavorable clinical outcomes across multiple malignancies [2,3].

The CSC state is not determined exclusively by stable genetic mutations but can be acquired, maintained, or lost through dynamic epigenetic and microenvironment-dependent processes [4,5,6]. Epigenetic regulation provides a potentially reversible mechanism through which cancer cells adapt their transcriptional states to environmental stress and therapeutic pressure [4,5]. DNA methylation, histone modification, chromatin remodeling, and non-coding RNA-mediated regulation influence the balance between self-renewal and differentiation, epithelial–mesenchymal plasticity, quiescence, and therapy-induced cellular reprogramming [4,5,6]. Importantly, differentiated cancer cells may reacquire stem-like properties under appropriate microenvironmental or treatment-induced conditions, thereby replenishing the CSC pool and contributing to intratumoral heterogeneity [2,4,5,6]. Epigenetic plasticity therefore represents an important mechanistic link among cancer stemness, metastatic competence, tumor evolution, and acquired drug resistance [4,5,6].

At the molecular level, CSC plasticity is regulated by interconnected epigenetic mechanisms involving DNA methyltransferases (DNMTs), histone-modifying enzymes, Polycomb complexes, and non-coding RNAs (ncRNAs) [5,6,7]. DNMT1 preserves DNA-methylation patterns during replication and can maintain transcriptional states that favor stem-cell persistence; direct genetic and pharmacological evidence in mammary tumor models has shown that DNMT1 supports CSC maintenance and that its inhibition restricts the CSC pool [8]. Histone deacetylases, including HDAC1 and HDAC2, remove acetyl groups from histone and non-histone proteins and are commonly associated with reduced chromatin accessibility and repression of differentiation-related or tumor-suppressive programs [5,6]. Enhancer of zeste homolog 2 (EZH2), the catalytic component of Polycomb Repressive Complex 2, deposits the repressive H3K27me3 mark and has been implicated in the expansion, maintenance, and therapeutic resistance of CSC populations in several malignancies [6,9]. In parallel, microRNAs (miRNAs), long non-coding RNAs (lncRNAs), and circular RNAs (circRNAs) form regulatory networks that modulate CSC self-renewal, differentiation, epithelial–mesenchymal transition (EMT), and treatment response [7]. Collectively, these mechanisms influence pluripotency-associated transcription factors such as SOX2, OCT4, NANOG, and KLF4; commonly used CSC-associated markers such as CD44, CD133, and ALDH1; and EMT regulators including ZEB1/2, SNAIL, SLUG, and TWIST [5,6,7].

Among naturally occurring bioactive compounds, curcumin, a polyphenolic constituent of the rhizome of Curcuma longa, has attracted considerable interest because of its ability to influence multiple cancer-associated signaling and transcriptional pathways [9]. Curcumin has been extensively investigated in preclinical models for its antiproliferative, pro-apoptotic, anti-inflammatory, antioxidant, and chemosensitizing activities [9]. Curcumin (diferuloylmethane) has been investigated as a pleiotropic small molecule capable of influencing inflammatory, redox, survival, apoptotic, angiogenic, and stress-response pathways rather than as a highly selective single-target agent [10,11,12,13,14,15,16,17,18,19,20]. Early dose-escalation and oncology studies generally reported oral tolerability, but they also exposed major translational limitations, including poor aqueous solubility, chemical instability, rapid conjugation, and low systemic bioavailability [21,22,23]. Medicinal-chemistry analyses have further cautioned that curcumin’s reactivity and assay-interference potential can complicate mechanistic attribution [24,25,26]. These broad network effects and pharmacological limitations are directly relevant to the present review: the former provide a rationale for coordinated modulation of DNA methylation, chromatin regulation, and non-coding RNA circuits, whereas the latter demand stringent causal validation in prospectively defined cancer stem-cell models. Accordingly, this review does not re-examine the entire anticancer literature on curcumin; it focuses on whether epigenetic changes induced by curcumin can plausibly destabilize CSC plasticity, therapeutic persistence, and relapse-associated phenotypes.

**Figure 1:**
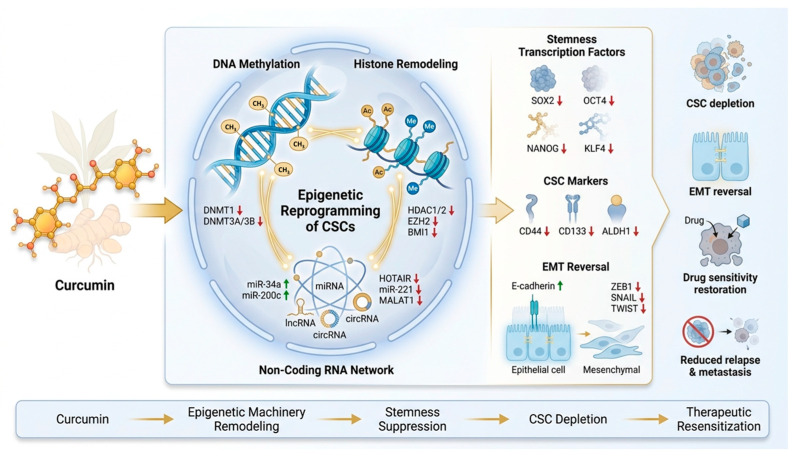
Evidence-based conceptual framework for curcumin-mediated epigenetic modulation of CSC plasticity. Studies conducted in different cancer models indicate that curcumin can modulate selected DNA methyltransferases, chromatin regulators, and non-coding RNAs. These changes may converge on transcriptional programs controlling stemness, epithelial–mesenchymal plasticity, and treatment response. The representation integrates direct molecular observations with proposed downstream CSC consequences; it does not imply that all targets are altered simultaneously or that CSC depletion has been causally demonstrated in a single model. Solid connectors indicate directly demonstrated curcumin-associated effects, whereas dashed connectors indicate proposed or independently supported links.

In addition to these conventional effects, experimental and computational studies indicate that curcumin can modulate epigenetic regulation by influencing DNMT activity, histone acetyltransferases and deacetylases, Polycomb-associated proteins, and ncRNA expression [27,28,29,30,31,32]. Curcumin has been reported to inhibit DNMT activity and alter DNA-methylation patterns [29,30], reduce EZH2 expression in defined cancer-cell models [31], and modify miRNA-expression profiles in cancer cells [29,32]. Nevertheless, these findings arise from different tumor types, experimental systems, curcumin concentrations, and treatment conditions; therefore, they do not yet establish a universal causal pathway linking curcumin-induced epigenetic remodeling directly to CSC eradication. Rather, the available evidence supports the testable hypothesis that coordinated modulation of these epigenetic regulators may weaken the molecular programs that sustain CSC plasticity, EMT, and therapeutic resistance. The proposed mechanistic framework linking curcumin-mediated epigenetic remodeling to stemness suppression, CSC depletion, and enhanced therapeutic sensitivity is summarized in Figure 1.

Previous reviews have examined the general epigenetic actions of curcumin [27,28,29], the epigenetic regulation of CSC formation and maintenance [5,6,7], and the ability of curcumin to affect CSC-associated signaling and survival [33,34,35]. However, a focused synthesis integrating curcumin-mediated regulation of DNA methylation, histone remodeling, and ncRNA networks specifically within the context of CSC plasticity remains limited. This review therefore evaluates the evidence supporting curcumin as a multi-layered epigenetic modulator of cancer stemness, while distinguishing direct experimental findings from proposed mechanistic connections. Particular attention is given to DNMT-dependent methylation, HDAC- and Polycomb-mediated chromatin regulation, and ncRNA circuits that influence stemness, EMT, tumor recurrence, and treatment resistance. Through this evidence-based framework, we examine the potential and current limitations of curcumin-mediated epigenetic reprogramming as a strategy for weakening CSC-associated phenotypes and improving therapeutic responsiveness.

### 1.1. Literature Search Strategy and Study Selection

This article was prepared as a structured critical narrative review. PubMed/MEDLINE, Scopus, and the Web of Science Core Collection were searched from database inception to 30 June 2026, which was also the date of the final literature search. The relevant literature was identified through searches of PubMed/MEDLINE, Scopus, and the Web of Science Core Collection. Google Scholar and the reference lists of relevant original studies and review articles were used for supplementary citation tracking. Search terms were combined with the Boolean operators AND and OR and included “curcumin” OR “curcuminoid”; “cancer stem cell” OR “tumor-initiating cell” OR stemness OR tumorsphere OR self-renewal; epigenetic OR “DNA methylation” OR DNMT1 OR DNMT3A OR DNMT3B OR “histone modification” OR “histone acetylation” OR HDAC OR EZH2 OR H3K27me3 OR BMI1 OR Polycomb OR “non-coding RNA” OR microRNA OR miRNA OR lncRNA OR circRNA; and plasticity OR “epithelial–mesenchymal transition” OR metastasis OR recurrence OR relapse OR “drug resistance” OR chemosensitization. Targeted searches additionally used miR-34a, miR-200c, miR-21, miR-221/222, H19, HOTAIR, MALAT1, circHN1, CD44, CD133, ALDH1, SOX2, OCT4, and NANOG.

Priority was given to peer-reviewed original studies that evaluated native curcumin or a clearly specified curcumin formulation, measured an epigenetic target, and reported a stemness-, plasticity-, resistance-, or tumor-initiation-related endpoint. Reviews were used for conceptual background and citation discovery, and selected earlier landmark studies were retained when mechanistically necessary. Studies of curcumin analogues were included only when the analogue was explicitly identified and were not treated as evidence for native curcumin. Evidence generated in bulk cancer-cell populations was distinguished from evidence obtained in prospectively isolated CSCs, tumorspheres, patient-derived organoids, xenografts, serial transplantation, or limiting-dilution tumor-initiation assays. Because the purpose was critical mechanistic synthesis rather than exhaustive quantitative pooling, no meta-analysis was performed.

To formalize the evidence-weighted synthesis, individual mechanistic claims were rated as Strong, Moderate, Limited, or Very limited/hypothesis-generating. Strong evidence required direct curcumin exposure in prospectively defined CSCs, confirmation of the proposed epigenetic alteration, regulator-specific rescue or loss-of-function testing, and a rigorous functional CSC endpoint such as serial self-renewal or limiting-dilution tumor initiation. Moderate evidence required direct curcumin-associated epigenetic regulation and a relevant stemness, plasticity, or resistance phenotype in the same experimental system, with partial causal validation. Limited evidence comprised direct curcumin–epigenetic findings in bulk cancer cells or independent CSC evidence that had not been causally integrated within the same model. Very limited/hypothesis-generating evidence included association studies, analogue- or formulation-dependent observations, conflicting findings, or links inferred across separate experimental systems. Because the included evidence was predominantly heterogeneous and preclinical, conventional GRADE was not applied directly; however, the framework incorporated the principles of directness, consistency, methodological limitations, and certainty of inference [36].

### 1.2. Use of Generative Artificial Intelligence

During the preparation of this work, all literature searches, critical evaluation of studies, data synthesis, interpretation of findings, and initial drafting of the manuscript were performed manually by the authors. The fundamental concepts and scientific conclusions presented in this review were derived solely from the authors’ independent analysis of the published literature. ChatGPT (Version: GPT-5.6 Sol, OpenAI, San Francisco, CA, USA) was subsequently used solely to improve language quality, simplify sentence structure, and enhance the clarity and readability of the manuscript, including assisting in the presentation of complex mechanistic concepts in a more accessible manner for readers. After using this tool, the authors reviewed and edited the content as needed and took full responsibility for the publication’s content.

## 2. Curcumin-Mediated Regulation of DNA Methylation in Cancer Stem Cells

### Mechanistic Basis and Current Evidence

Curcumin-mediated DNA methylation is a covalent epigenetic modification involving the addition of a methyl group to the fifth carbon of cytosine, predominantly within CpG dinucleotides. This reaction is catalyzed by DNMTs, among which DNMT1 preferentially preserves existing methylation patterns during DNA replication, whereas DNMT3A and DNMT3B participate mainly in the establishment of new methylation patterns [4,5,6]. In normal tissues, DNA methylation contributes to genomic stability, lineage identity, and transcriptional regulation. Cancer cells, however, commonly exhibit focal hypermethylation of promoter-associated CpG islands together with broader genomic hypomethylation, resulting in the silencing of tumor-suppressive programs and destabilization of the cancer epigenome [4,5,6,37]. These alterations can support epithelial–mesenchymal plasticity, drug tolerance, and the acquisition or maintenance of stem-like cellular states. Experimental studies in mammary, pancreatic, esophageal, and liver cancer models demonstrate that DNMT-dependent methylation is functionally involved in CSC self-renewal and tumorigenicity rather than merely being associated with these phenotypes [8,38,39,40,41].

Among the DNMT family, the evidence linking DNMT1 to CSC maintenance is particularly strong. Genetic depletion or pharmacological inhibition of DNMT1 reduced mammary CSC formation and tumorigenic capacity in experimental models [8]. Pancreatic CSCs similarly displayed increased dependence on DNMT1, and DNMT1 inhibition reduced their self-renewal and reprogrammed them toward a less stem-like state through epigenetic reactivation of the miR-17-92 cluster [38]. In esophageal squamous cell carcinoma, DNMT1 was enriched in side-population and tumorsphere-forming cells, whereas DNMT1 knockdown impaired self-renewal and tumor formation [39]. Liver cancer studies further demonstrated that DNMT1-dependent methylation of BEX1 or SOCS1 contributes to the maintenance of stemness and tumorigenicity [40,41]. These observations establish DNMT1 as an important regulator of CSC identity in defined experimental systems. They do not, however, demonstrate that DNMT1 directly controls every commonly used CSC marker, including CD44, CD133, SOX2, OCT4, and NANOG, across all tumor types.

Curcumin has been reported to influence DNA methylation through both enzymatic and transcriptional mechanisms. Biochemical and molecular-modeling experiments suggested that curcumin can inhibit DNMT1 catalytic activity [30]. In acute myeloid leukemia cells, primary leukemic blasts, and a xenograft model, curcumin reduced DNMT1 expression by suppressing the DNMT1 transcriptional regulators NF-κB/p65 and Sp1. This reduction was accompanied by hypomethylation and re-expression of p15INK4B, G1 cell-cycle arrest, apoptosis, and inhibition of tumor growth [42]. In breast cancer cells, curcumin reduced DNMT1 expression and methyltransferase activity and partially reactivated the methylation-silenced tumor suppressor RASSF1A [43]. These studies provide direct evidence that curcumin can affect DNMT1 and restore selected epigenetically silenced genes, although neither study examined purified CSC populations nor demonstrated that DNMT1 inhibition was solely responsible for CSC depletion.

Importantly, curcumin should not be described as a universal or indiscriminate DNA-demethylating agent. Genome-wide analysis in colorectal cancer cells found no generalized reduction in global DNA methylation after curcumin treatment. Instead, prolonged exposure produced both hypermethylation and hypomethylation at selected, predominantly partially methylated CpG loci, with substantial variation among cell lines [37]. Some of these methylation alterations correlated with changes in gene expression, suggesting a locus- and context-dependent effect rather than global demethylation [37]. Additional studies reported curcumin-associated demethylation and re-expression of specific genes, including p21Cip1 and BRCA1, in defined cancer-cell models [44,45]. Conversely, another breast cancer study found that although curcumin reduced DNMT1, DNMT3A, and DNMT3B expression, it did not alter the methylation status of the particular promoter panel examined [46]. Therefore, decreased DNMT expression should not automatically be interpreted as evidence of promoter demethylation or tumor-suppressor reactivation.

Evidence concerning DNMT3A and DNMT3B is less developed and remains highly model-dependent. Dendrosomal nanocurcumin reduced DNMT1, DNMT3A, and DNMT3B expression while increasing miR-34 family expression in HepG2 and Huh7 hepatocellular carcinoma cells [47]. In non-small-cell lung cancer cells, curcumin reduced the expression of all three DNMTs, restored miR-142-5p expression, and increased sensitivity to crizotinib through inhibition of ULK1-dependent autophagy [48]. In gastric cancer cells, curcumin-induced DNA demethylation was linked to a DNA-damage-response pathway involving p53, p21/GADD45A, cell-cycle regulators, E2F, and DNMT1 [49]. These findings support the capacity of curcumin to alter the DNA-methylation machinery and epigenetically regulated transcription in specific experimental contexts. However, direct evidence that curcumin restores PTEN, CDH1, p16INK4A, or TP53 specifically through DNMT3A/3B inhibition in CSCs is currently insufficient.

**Figure 2:**
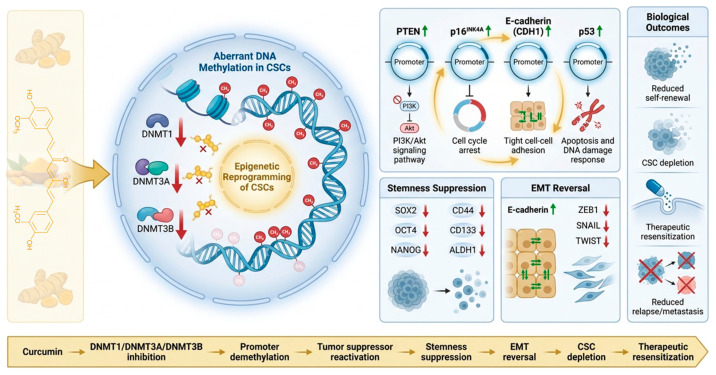
Proposed contribution of curcumin-mediated DNA-methylation remodeling to reduced CSC plasticity. Curcumin has altered DNMT1 expression or activity and selected locus-specific methylation states in defined cancer models, whereas independent studies establish roles for DNMT-dependent methylation in CSC maintenance. The proposed convergence on tumor-suppressive, epithelial, and stemness-related programs remains context dependent and requires validation in prospectively isolated CSCs using locus-specific methylation analysis and genetic rescue. The diagram should not be interpreted as evidence that curcumin universally demethylates PTEN, CDH1, CDKN2A, or TP53 through DNMT3A/3B inhibition in CSCs.

Taken together, DNMT1-dependent methylation supports CSC maintenance in several tumor models [8,38,39,40,41], whereas curcumin can alter DNMT activity, expression, and locus-specific methylation in cancer cells [30,37,42,43,44,45,46,47,48,49]. These complementary findings support, but do not yet establish, a causal curcumin–DNMT–CSC axis. Validation requires CSC-resolved methylation profiling combined with DNMT rescue and functional self-renewal or tumor-initiation assays. Figure 2 therefore presents a context-dependent mechanistic model rather than a universal demethylation pathway.

## 3. Curcumin and the Histone Remodeling Machinery in Cancer Stem Cells

Beyond DNA methylation, covalent histone modifications and Polycomb-mediated chromatin regulation provide an additional layer of epigenetic control over cancer stem cell (CSC) plasticity. Histone acetylation, methylation, and other post-translational modifications influence the recruitment of regulatory proteins and the accessibility of chromatin to the transcriptional machinery [5,6,50]. Histone acetylation is commonly associated with accessible chromatin, whereas the transcriptional consequences of histone methylation depend on the modified residue, the degree of methylation, and the surrounding chromatin context [50]. By dynamically altering these states, cancer cells can maintain self-renewal programs, suppress differentiation, undergo epithelial–mesenchymal plasticity, and adapt to therapeutic stress [5,6]. Histone-modifying enzymes are therefore important regulators of CSC identity, although their functions are tumor- and context-dependent.

Histone deacetylases (HDACs) remove acetyl groups from histone and non-histone proteins and participate in multiprotein complexes that regulate transcription, DNA repair, cell-cycle progression, and cellular differentiation [50]. Aberrant activity of class I HDACs, including HDAC1, HDAC2, and HDAC3, has been associated with malignant progression and stem-like phenotypes in several experimental systems [5,6]. Nevertheless, HDAC inhibition should not automatically be equated with CSC depletion. In human breast cancer models, pharmacological HDAC inhibition promoted dedifferentiation and increased stem-like properties through activation of WNT/β-catenin signaling [51]. Thus, the biological consequences of targeting HDACs depend on the HDAC isoform, tumor lineage, treatment schedule, and cellular state.

Direct studies indicate that curcumin can modulate HDAC expression and activity, but the affected isoforms vary among experimental models. In Raji B-cell lymphoma cells, curcumin reduced the expression of HDAC1, HDAC3, and HDAC8, increased acetylated histone H4, inhibited proliferation, and induced apoptosis [52]. In medulloblastoma models, curcumin reduced total HDAC activity and HDAC4 expression, increased acetylation of the non-histone substrate tubulin, induced G2/M arrest and apoptosis, and restricted tumor growth in vivo [53]. These findings support HDAC-related actions of curcumin but do not establish universal inhibition of HDAC1, HDAC2, and HDAC3. Moreover, curcumin can also inhibit the histone acetyltransferase activity of p300/CBP and reduce the acetylation of histone and non-histone substrates [54]. Curcumin should therefore be regarded as a context-dependent regulator of the acetylation–deacetylation balance rather than as a compound that uniformly increases histone acetylation. Importantly, the cited HDAC studies did not test whether HDAC modulation was necessary or sufficient for curcumin-induced depletion of purified CSC populations.

EZH2, the catalytic component of Polycomb Repressive Complex 2, catalyzes the mono-, di-, and trimethylation of histone H3 at lysine 27, with H3K27me3 commonly functioning as a transcriptionally repressive chromatin mark [50,55]. EZH2 contributes to CSC biology through both canonical chromatin-dependent and non-canonical signaling mechanisms. In breast cancer, EZH2 promoted expansion of tumor-initiating cells through a RAF1–ERK–β-catenin signaling mechanism [56]. In hepatocellular carcinoma models, pharmacological depletion of EZH2 and H3K27me3 by 3-deazaneplanocin A impaired tumor-initiating cell properties and tumorigenicity [57]. These studies directly support EZH2 as a regulator of CSC maintenance, although the downstream genes and signaling mechanisms vary among cancer types.

Curcumin has been shown to suppress EZH2-related signaling in several non-CSC experimental systems. In MDA-MB-435 cells, curcumin reduced EZH2 expression through MAPK-dependent regulation [31]. In myelodysplastic syndrome-derived cell lines and xenografts, curcumin decreased EZH2 and H3K27me3 levels, inhibited their nuclear localization, induced cell-cycle arrest and apoptosis, and reduced tumor growth [58]. In lung cancer cells, curcumin suppressed EZH2 transcription and mRNA stability, partly through induction of miR-101 and let-7c, and attenuated the reciprocally regulated EZH2–NOTCH1 pathway [59]. These studies demonstrate that curcumin can regulate EZH2 expression and, in some models, reduce H3K27me3. However, they do not directly establish that curcumin reactivates CDH1, p21, and PTEN through EZH2 inhibition in CSCs, nor do they prove that EZH2 suppression is required for curcumin-mediated CSC depletion.

BMI1, a core component of canonical Polycomb Repressive Complex 1, represents another epigenetic regulator associated with malignant stem-cell persistence. BMI1 contributes to transcriptional repression, self-renewal, survival, and therapeutic resistance by regulating chromatin states and senescence-associated pathways. In head and neck squamous cell carcinoma, BMI1 expression was enriched in CSC populations and functionally linked to sphere formation and cisplatin resistance; suppression of the IL-6R–BMI1 axis reduced the CSC fraction and impaired self-renewal [60]. Curcumin has been reported to reduce BMI1 protein expression in colorectal cancer cells [61]. Nevertheless, that study did not use purified CSC populations or demonstrate through BMI1 rescue experiments that reduced BMI1 expression mediated the effects of curcumin on SOX2, OCT4, NANOG, self-renewal, or therapeutic sensitivity.

**Figure 3:**
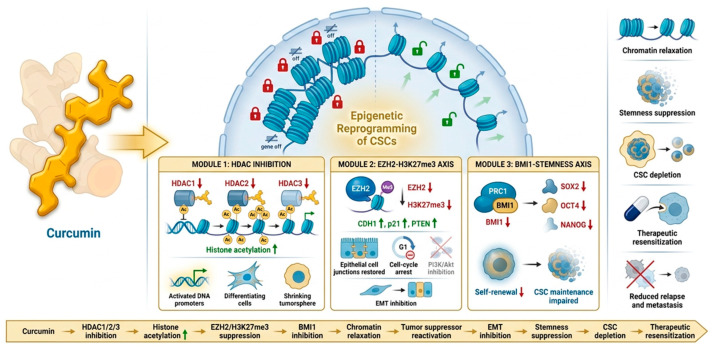
Conceptual model linking curcumin-associated chromatin regulation to CSC plasticity. Curcumin has modulated selected HDAC isoforms, p300/CREB-binding protein activity, EZH2/H3K27me3, and BMI1 in separate experimental systems. Because curcumin can either increase or decrease acetylation depending on the target and context, it should be described as a regulator of the acetylation–deacetylation balance rather than a uniform HDAC inhibitor. Downstream effects on CSC self-renewal, epithelial–mesenchymal plasticity, and therapeutic response are proposed and require isoform-specific depletion/rescue and tumor-initiation studies.

Overall, curcumin modulates selected HDAC isoforms, p300/CBP, EZH2/H3K27me3, and BMI1 in model-specific settings [31,52,53,54,58,59,61]. Independent studies implicate these regulators in CSC behavior [5,6,51,56,57,60], but the two evidence streams have rarely been tested together. Future studies should combine CSC-resolved chromatin profiling with regulator-specific depletion or rescue and functional tumor-initiation assays. Figure 3 and Table 1 summarize these mechanisms and their level of causal support.

## 4. Curcumin-Mediated Modulation of Non-Coding RNA Networks Governing Cancer Stem Cell Plasticity

In addition to DNA methylation and histone modification, ncRNAs provide an important regulatory layer governing cancer stem cell (CSC) plasticity. MicroRNAs (miRNAs) primarily regulate gene expression post-transcriptionally, whereas lncRNAs and circular RNAs (circRNAs) can influence transcription, RNA stability, translation, chromatin organization, and interactions among regulatory RNAs [5,6,7]. Through these mechanisms, ncRNA networks contribute to self-renewal, epithelial–mesenchymal plasticity, metabolic adaptation, and therapeutic resistance [7]. Curcumin has been shown to alter miRNA-expression profiles in cancer cells, providing initial evidence that ncRNA modulation may contribute to its anticancer activity [32]. However, these effects vary according to the tumor type, curcumin formulation, dose, and experimental context.

miR-34a represents one of the strongest mechanistic links between ncRNA regulation and CSC behavior. In prostate cancer, miR-34a was underexpressed in purified CD44-positive CSC-enriched populations, whereas enforced miR-34a expression suppressed clonogenic expansion, tumor regeneration, and metastasis by directly targeting CD44 [62]. In colon cancer stem cells, miR-34a acted as a cell-fate determinant by modulating Notch signaling and the balance between self-renewal and differentiation [63]. Direct evidence also connects curcumin with the miR-34 family. In colorectal cancer cells, curcumin activated a ROS–KEAP1–NRF2 pathway that induced miR-34a and miR-34b/c independently of p53 [64]. Genetic deletion of these miRNAs attenuated curcumin-induced apoptosis and senescence and reduced its inhibitory effects on migration and invasion. Curcumin also promoted a mesenchymal-to-epithelial transition and suppressed experimental lung metastasis in a miR-34a-dependent manner [64]. Nevertheless, this study did not prospectively isolate CSCs or directly demonstrate that curcumin-induced miR-34a expression depletes CSCs or abolishes tumorsphere formation. The proposed curcumin–miR-34a–CSC connection therefore combines direct curcumin evidence with independent CSC-specific evidence rather than representing a fully validated causal pathway.

The miR-200 family is a major regulator of epithelial identity and epithelial–mesenchymal transition (EMT). miR-200 family members directly target the E-cadherin repressors ZEB1 and ZEB2, thereby promoting epithelial gene expression and limiting migratory behavior [65]. In colorectal cancer cells, curcumin increased miR-200c expression, and miR-200c induction was required for curcumin-mediated inhibition of EMT, migration, and invasion [66]. In that study, EPM5, rather than ZEB1 or ZEB2, was experimentally validated as the critical direct target of miR-200c mediating the effects of curcumin [66]. A separate study of 5-fluorouracil-resistant colorectal cancer cells showed that curcumin increased several EMT-suppressive miRNAs, including miR-200c, reduced EMT-associated and Polycomb-related factors, and restored sensitivity to 5-fluorouracil in cellular and xenograft models [67]. These findings provide strong evidence for curcumin-mediated miRNA-dependent suppression of EMT and chemoresistance. However, neither study directly established depletion of prospectively defined CSC populations through a miR-200c–ZEB1/2 pathway.

Curcumin also modulates the oncogenic miRNA miR-21 in several cancer models. In colorectal cancer cells, curcumin inhibited AP-1-dependent miR-21 transcription, increased the tumor suppressor PDCD4, and reduced invasion and experimental metastasis [68]. In MCF-7 breast cancer cells, curcumin decreased miR-21, increased PTEN, reduced Akt phosphorylation, and inhibited proliferation; experimentally increasing miR-21 attenuated these effects [69]. Similar concentration-dependent decreases in miR-21, increases in PTEN, and inhibition of phosphorylated Akt were observed in gastric cancer cells [70]. These studies support curcumin regulation of the miR-21–PDCD4 and miR-21–PTEN–Akt axes. Because PTEN/Akt- and PDCD4-associated pathways influence survival, EMT, and therapy response, these mechanisms may be relevant to CSC persistence. Nevertheless, the cited studies used conventional cancer-cell populations rather than isolated CSCs, and they do not establish curcumin-mediated therapeutic resensitization specifically through miR-21 suppression in CSCs.

The evidence involving miR-221 requires particular caution. In pancreatic cancer cells, miR-221 inhibition increased PTEN, p27Kip1, p57Kip2, and PUMA and reduced proliferation [71]. However, the compound used to suppress miR-221 in that study was CDF, a synthetic curcumin analogue, rather than native curcumin [71]. More recently, a chitosan–cyclodextrin curcumin nanoformulation reduced miR-221 and miR-222 expression in MCF-7 and MDA-MB-231 breast cancer cells, but the response differed in SK-BR-3 cells [72]. These findings indicate formulation- and cell-type-dependent regulation of miR-221/222. They do not support a universal pathway in which unformulated curcumin suppresses miR-221, restores PTEN and p27, and depletes CSCs.

Evidence for curcumin-mediated lncRNA regulation is also emerging but remains uneven. In gastric cancer cells, curcumin reduced H19 expression, and experimental H19 overexpression partially reversed curcumin-induced growth inhibition, supporting a functional curcumin–H19 relationship [73]. In tamoxifen-resistant breast cancer cells, curcumin attenuated H19-associated EMT, migration, and invasion and restored epithelial characteristics [74]. Curcumin also inhibited the migration of renal cell carcinoma cells with different levels of HOTAIR expression, suggesting that HOTAIR may influence cellular responsiveness to curcumin; however, that study did not establish that curcumin directly suppresses HOTAIR expression [75]. Similarly, silencing MALAT1 increased the inhibitory effects of curcumin on the viability, migration, and invasion of SW480 colorectal cancer cells, but this finding demonstrates cooperation between MALAT1 knockdown and curcumin rather than direct downregulation of MALAT1 by curcumin [76].

Curcumin-mediated circRNA regulation has also been demonstrated in selected non-CSC models. In colorectal cancer cells, curcumin reduced circHN1 expression and inhibited proliferation, colony formation, migration, invasion, and EMT through a circHN1–miR-302a-3p–PIK3R3 regulatory axis [77]. Restoration of circHN1 partially reversed several effects of curcumin, providing mechanistic evidence for the involvement of this circRNA pathway [77]. Nevertheless, direct studies connecting curcumin-regulated circRNAs with CSC self-renewal, tumor initiation, or therapeutic resistance remain scarce. The ncRNA evidence is consolidated in Table 2 to distinguish direct curcumin–ncRNA experiments from independent evidence linking the same ncRNA to CSC behavior.

**Figure 4:**
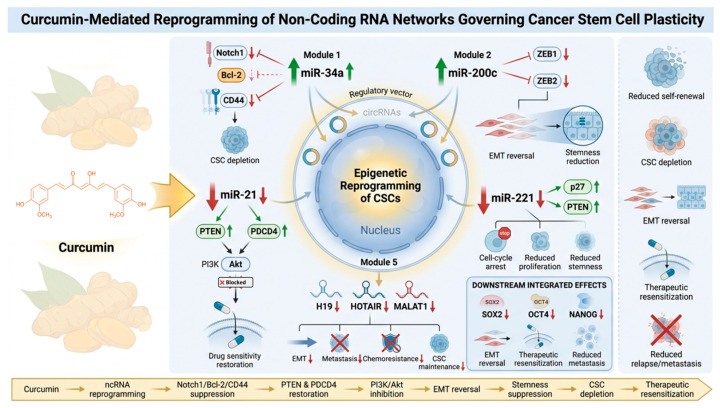
Evidence-weighted network of curcumin-responsive non-coding RNAs relevant to CSC-associated phenotypes. Direct curcumin-associated regulation is best supported for the miR-34 family, miR-200c, miR-21, H19, and circHN1 in defined cancer models. Evidence involving miR-221/222 depends on a curcumin analogue or specialized formulation, whereas the HOTAIR and MALAT1 studies demonstrate response modification or combination effects rather than consistent direct downregulation by native curcumin. Proposed effects on CSC depletion and relapse should therefore be shown as inferential rather than established causal outcomes.

Collectively, current evidence demonstrates that curcumin can modulate selected tumor-suppressive and oncogenic ncRNAs, including the miR-34 family, miR-200c, miR-21, H19, and circHN1, in defined cancer models [64,66,67,68,69,70,73,74,77]. More limited evidence implicates miR-221, HOTAIR, and MALAT1, but these findings depend on curcumin analogues, specialized formulations, or combination experiments and should not be interpreted as universal effects of native curcumin [71,72,75,76]. Independent studies establish that several of these ncRNAs regulate CSC self-renewal, differentiation, EMT, and tumor initiation [7,62,63]. Therefore, ncRNA modulation provides a biologically plausible mechanism through which curcumin may weaken CSC-associated plasticity and therapeutic resistance. However, the integrated sequence of curcumin-induced ncRNA reprogramming, CSC depletion, and therapeutic resensitization has not yet been demonstrated as a single causal pathway. Figure 4 should consequently be interpreted as an evidence-based conceptual network that integrates direct experimental findings with proposed CSC-related consequences.

## 5. Integrated Model: From Epigenetic Reprogramming to Suppression of Cancer Stem Cell Plasticity

The evidence reviewed above supports a multi-layered model in which DNA methylation, histone and Polycomb regulation, and non-coding RNA networks collectively contribute to cancer stem cell (CSC) plasticity. These regulatory systems interact with signaling and transcriptional programs governing self-renewal, differentiation, epithelial–mesenchymal plasticity, quiescence, and treatment adaptation [4,5,6,7,8,55]. Direct experimental studies have established functional roles for DNMT1, EZH2, BMI1, miR-34a, and related epigenetic regulators in the maintenance of stem-like cancer populations in selected tumor models [8,38,39,40,41,56,57,60,62,63]. However, the relative importance of each mechanism differs among tumor types, cellular states, and therapeutic conditions. CSC persistence should therefore be understood as an adaptive network state rather than the product of one epigenetic lesion or signaling pathway. Because the proposed epigenetic-collapse model is hypothesis-generating, Table 3 defines the minimum evidence needed to establish causal curcumin-mediated reprogramming of CSC plasticity.

The application of this framework showed that no complete curcumin–epigenetic–CSC pathway currently meets the criteria for Strong evidence. The miR-34 and miR-200c axes reach Moderate evidence for selected molecular, EMT, metastatic, or treatment-response phenotypes but remain Limited for direct depletion of prospectively defined CSCs. Most DNMT-, HDAC/p300-, EZH2/BMI1-, lncRNA-, and circRNA-related links remain Limited or Very limited for CSC-specific outcomes because epigenetic target engagement, regulator-specific rescue, and rigorous functional CSC assays have not been integrated within the same experimental model. Separate lines of experimental evidence demonstrate that curcumin can influence several components of this network. Depending on the experimental model, curcumin has altered DNMT expression or locus-specific DNA methylation, modulated selected HDAC isoforms and acetyltransferase activity, reduced EZH2/H3K27me3 or BMI1 expression, and changed the abundance of tumor-suppressive and oncogenic ncRNAs [30,31,32,37,42,43,44,46,54,58,64,66,77]. Among the better-supported ncRNA effects are induction of the miR-34 family and miR-200c and repression of miR-21 [64,66,68,69,70]. Evidence for miR-221 regulation is more formulation- and cell-type-dependent [71,72]. These findings indicate that curcumin can act at multiple epigenetic levels, but they do not demonstrate that all of these targets are modified simultaneously or with comparable potency in the same CSC population.

The biological consequences of these epigenetic changes are also best interpreted as convergent rather than strictly linear. Curcumin-induced miR-34 signaling has been linked to inhibition of colorectal cancer migration, invasion, and experimental metastasis [64], whereas miR-200c-associated responses have been implicated in EMT suppression and restoration of sensitivity to 5-fluorouracil in colorectal cancer models [66,67]. Curcumin-mediated regulation of miR-21 has restored PDCD4 or PTEN-associated signaling and reduced invasive or proliferative phenotypes in defined cancer-cell systems [68,69,70]. In addition, targeted curcumin nanomicelles inhibited breast cancer-cell growth and tumorsphere formation in a breast CSC-enriched model [78]. These observations support the possibility that epigenetic and ncRNA modulation contributes to reduced stem-like behavior and improved treatment response. Nevertheless, most studies did not combine epigenetic target rescue, serial self-renewal analysis, and limiting-dilution tumor-initiation assays; therefore, epigenetic reprogramming cannot yet be identified as the exclusive cause of the observed CSC-associated effects.

**Figure 5:**
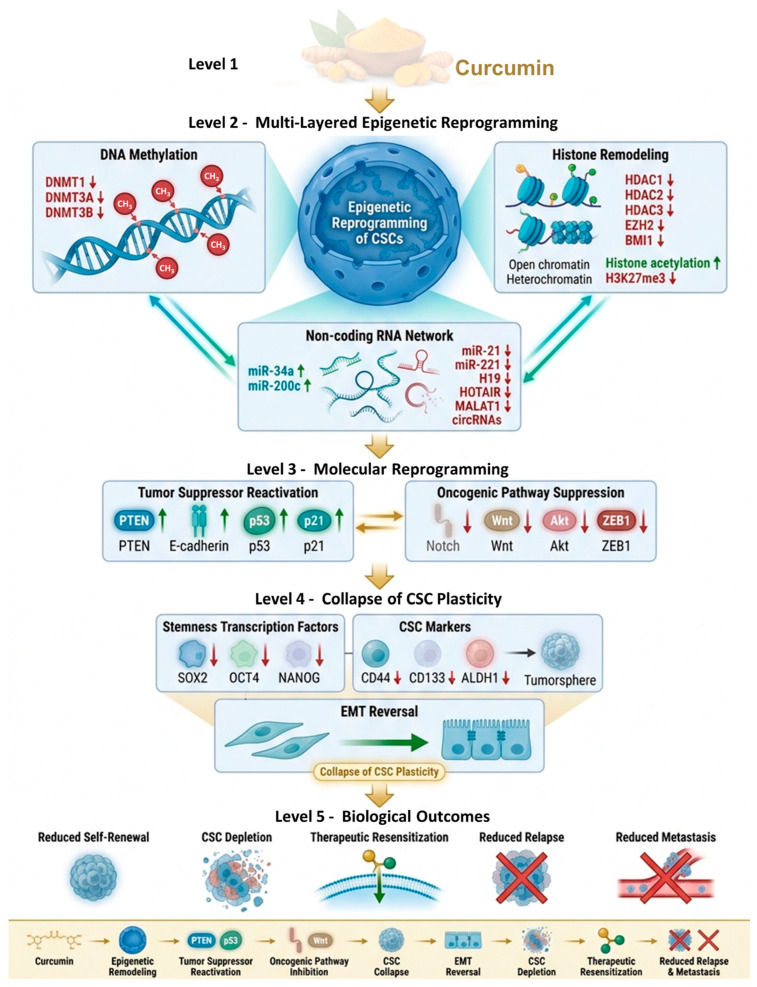
Proposed integrated model of curcumin-mediated epigenetic modulation of cancer stem cell plasticity. Curcumin may influence multiple interconnected epigenetic layers, including DNA methylation, histone and Polycomb regulation, and non-coding RNA networks. These changes may converge on tumor-suppressive and oncogenic pathways that regulate stemness-associated transcription factors, cancer stem cell markers, epithelial–mesenchymal plasticity, self-renewal, therapeutic response, relapse, and metastasis. The upward and downward arrows indicate reported or proposed directional changes compiled from different experimental models and do not imply that all effects occur simultaneously or have been causally validated within a single cancer stem cell system.

Accordingly, the integrated model proposed here should be viewed as an evidence-based synthesis rather than an established universal pathway. Curcumin-mediated changes in DNA methylation, chromatin regulation, and ncRNA expression may converge on tumor-suppressive, EMT-associated, and stemness-related networks, thereby restricting CSC plasticity, reducing tumor-repopulating capacity, and increasing susceptibility to therapy. Definitive validation will require CSC-resolved epigenomic profiling and genetic rescue experiments demonstrating that specific epigenetic alterations are necessary for curcumin-induced suppression of self-renewal and tumor initiation. Figure 5 integrates reported and inferred effects from distinct experimental models; the arrows indicate proposed directionality rather than a single validated causal pathway.

## 6. Future Perspectives

Translating curcumin-mediated epigenetic regulation into cancer therapy requires stronger mechanistic and pharmacological validation. Because curcumin is chemically reactive, unstable under some conditions, and capable of assay interference, future studies should use chemically characterized preparations, appropriate interference controls, pharmacologically relevant concentrations, and genetic loss- and gain-of-function experiments [22]. CSC-resolved methylome, chromatin-accessibility, histone-mark, and ncRNA profiling should be integrated with patient-derived organoids and limiting-dilution tumor-initiation assays to distinguish epigenetic reprogramming from nonspecific cytotoxicity. Poor systemic exposure remains a major translational limitation of conventional oral curcumin [79,80]. Nanoparticle formulations can improve pharmacokinetic exposure [81], and targeted nanomicelles have inhibited breast cancer-cell growth and tumorsphere formation preclinically [78]. However, selective delivery to CSC niches has not been demonstrated clinically. Future formulation studies should quantify free curcumin and relevant metabolites in tumors, include carrier-matched controls, and measure CSC frequency, tumor-initiation capacity, biodistribution, toxicity, and pharmacodynamic epigenetic effects.

Curcumin metabolism may further modify the proposed epigenetic-plasticity network. Curcumin undergoes reduction to tetrahydrocurcumin and other hydrogenated metabolites, in addition to extensive conjugation, and tetrahydrocurcumin has been detected following oral administration of curcumin formulations in humans [82]. These metabolites should not be assumed to reproduce the epigenetic target profile of parent curcumin. In a direct comparative analysis, tetrahydrocurcumin showed a different HDAC1- and PCAF-related inhibitory profile from curcumin and weaker antiproliferative activity, indicating divergent rather than pharmacologically equivalent effects [83]. Conversely, tetrahydrocurcumin influenced DNMT1-associated promoter methylation in a non-cancer ischemia model, demonstrating context-dependent epigenetic activity [84]. Current evidence therefore does not establish directly opposing curcumin-versus-tetrahydrocurcumin effects in CSCs; however, metabolism may weaken, preserve, or redirect individual components of the proposed network. Future studies should compare parent curcumin, tetrahydrocurcumin, conjugated metabolites, and formulation-specific metabolite mixtures at measured intratumoral exposures using CSC-resolved epigenetic and functional assays.

Normal tissue stem-cell safety also requires explicit evaluation because the epigenetic regulators implicated in CSC plasticity contribute to the maintenance and differentiation of normal stem and progenitor compartments. Experimental disruption of Dnmt1 impairs hematopoietic stem- and progenitor-cell self-renewal, niche retention, and multilineage output [85], whereas loss of DNA-methylation maintenance in intestinal stem cells causes crypt expansion, inappropriate persistence of stem-cell-associated transcriptional programs, and delayed epithelial differentiation [86]. Nonselective or excessive epigenetic reprogramming could therefore theoretically produce hematopoietic suppression or lineage abnormalities in the bone marrow and impaired crypt regeneration, epithelial differentiation, or barrier repair in the intestine. These risks have not been established for pharmacologically achievable curcumin exposure and should not be inferred directly from cancer-cell studies. Future safety studies should include normal human CD34-positive hematopoietic progenitor assays, matched normal intestinal organoids, lineage-differentiation and barrier-function endpoints, reversibility after treatment withdrawal, and tumor-to-normal exposure comparisons.

Drug-resistant CSC models have important clinical relevance because they enable investigation of the cellular states that persist during therapy, repopulate tumors after treatment withdrawal, and contribute to recurrence. Drug-resistant cancer models can also reveal adaptive mechanisms that connect therapeutic resistance with metastatic behavior; for example, EGFR-TKI-resistant lung cancer cells exhibited mitochondrial metabolic reprogramming and enhanced metastatic potential [87]. However, drug resistance, metabolic adaptation, or metastatic behavior alone should not be interpreted as evidence of CSC identity without functional self-renewal and tumor-initiation assays. Commonly used systems include chronically drug-selected cell lines, marker-enriched or tumorsphere-forming populations, resistant-cell-derived xenografts, and patient-derived organoids. However, each model captures only selected dimensions of clinical resistance. Drug-selected cell lines may reflect adaptation to a single agent and prolonged in-vitro culture rather than the heterogeneous treatment histories and pharmacological exposures observed in patients. Marker-based isolation and tumorsphere formation enrich for stem-like phenotypes but do not necessarily establish durable tumor-initiating capacity, whereas xenografts and organoids provide greater biological relevance but may incompletely reproduce immune, stromal, vascular, and metastatic-niche interactions. Consequently, curcumin-mediated epigenetic effects should be validated across complementary resistant CSC models and confirmed using serial self-renewal, limiting-dilution tumor initiation, treatment-withdrawal, and relapse-related endpoints before clinical significance is inferred [2,3,4,78].

Patient-derived tumor explants and patient-derived tumor organoids (PDTOs) may provide a translational bridge between established cancer-cell models and clinically observed treatment responses. An ex vivo tumor-explant platform that preserved the tumor architecture, cellular heterogeneity, and microenvironmental context demonstrated an association between experimental drug responses and clinical outcomes in independent patient cohorts [88]. PDTO studies in rectal cancer have similarly shown retention of molecular and histopathological characteristics of the corresponding tumors and concordance between ex vivo chemotherapy or radiation responses and responses observed in individual patients [89,90]. These platforms are nevertheless complementary rather than interchangeable. Tumor explants permit short-term treatment testing within relatively intact tissue, whereas PDTOs enable expansion and repeated experimental perturbation. Conventional epithelial organoid cultures, however, generally do not retain the complete stromal and immune microenvironment unless specialized culture systems are used [91]. Moreover, because both are ex vivo systems, they cannot reproduce whole-body pharmacokinetics, metabolism, or systemic toxicity. Therefore, studies of curcumin should combine patient-derived models with pharmacologically credible exposure measurements, confirmation of epigenetic target engagement, and functional CSC assays rather than relying solely on short-term cell-viability responses.

**Figure 6:**
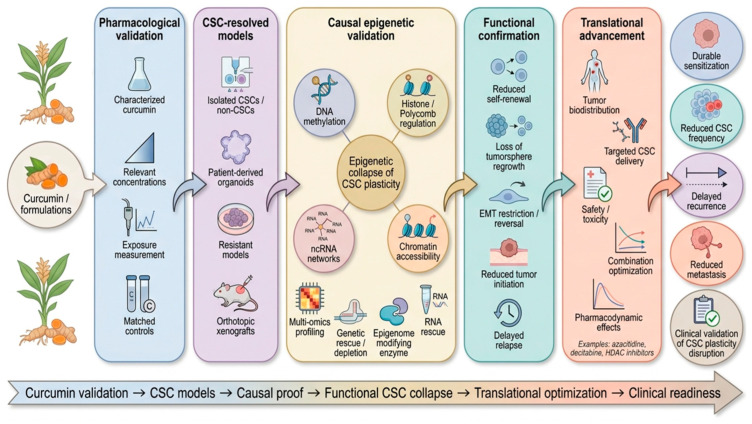
Future perspectives and translational roadmap for validating curcumin-induced epigenetic collapse of cancer stem cell plasticity. Future progress will require stepwise validation from pharmacological credibility and CSC-resolved model systems to causal epigenetic testing, functional confirmation, and translational development. Key priorities include characterized curcumin formulations, relevant exposure conditions, isolated CSC and non-CSC models, patient-derived and in vivo systems, and causal testing of DNA methylation, histone/Polycomb regulation, non-coding RNA networks, and chromatin accessibility. Functional studies should confirm reduced self-renewal, impaired tumorsphere regrowth, restricted EMT plasticity, reduced tumor initiation, and delayed relapse. Translational studies should then address biodistribution, targeted CSC delivery, safety, combination strategies, and pharmacodynamic effects to determine whether this framework can advance toward clinically relevant therapeutic sensitization and reduced recurrence.

Combination therapy also warrants investigation. Curcumin enhanced azacitidine activity in myeloid leukemia models and patient-derived samples [92], showed activity with azacitidine in decitabine-resistant colorectal cancer cells [93], and increased the effects of vorinostat and panobinostat through Hsp90-associated mechanisms [94]. These findings remain preclinical and require formal synergy analysis, exposure–response assessment, treatment-sequence optimization, and safety evaluation. Curcumin should therefore be considered an investigational adjuvant rather than a standalone epigenetic therapy. These priorities are summarized in Figure 6, which outlines a stepwise translational roadmap linking pharmacological validation and CSC-resolved model systems with causal epigenetic testing, functional confirmation of impaired CSC plasticity, and subsequent translational advancement toward clinical readiness.

## 7. Conclusions

By integrating previously fragmented evidence across DNA methylation, chromatin and Polycomb regulation, and non-coding RNA networks, this review advances a unified, evidence-weighted framework for understanding how curcumin may interfere with the epigenetic circuitry that sustains CSC plasticity. The principal conceptual contribution of this synthesis is the proposed “epigenetic collapse of CSC plasticity”, in which convergent perturbation of multiple epigenetic regulatory layers may reduce the capacity of cancer cells to maintain or reacquire stem-like states, undergo epithelial–mesenchymal state transitions, and adapt to therapeutic pressure. Current evidence supports curcumin as a context-dependent, multilayer epigenetic modulator; however, simultaneous reprogramming of these regulatory layers within the same prospectively defined CSC population has not yet been demonstrated. Accordingly, the proposed epigenetic-collapse model should be regarded as a testable mechanistic framework rather than an established universal pathway or evidence of CSC eradication. Its broader significance lies in shifting the focus from individual curcumin-responsive targets toward the integrated epigenetic networks that enable CSC adaptability and therapeutic persistence. Future validation in CSC-resolved and patient-derived models, combined with causal epigenetic interrogation and pharmacologically credible exposure, will determine whether disruption of this circuitry can produce durable reductions in tumor-initiating capacity, treatment resistance, and relapse.

## Figures and Tables

**Table 1:** Experimental evidence linking curcumin to DNA-methylation and chromatin-remodeling mechanisms relevant to CSC plasticity.

Model/Axis	Intervention	Direct Epigenetic Finding	Reported Biological Implication	Evidence Rating and CSC Interpretation	Ref.
DNMT1; biochemical/modeling system	Native curcumin	Inhibition of DNMT1 catalytic activity was proposed	Supports a direct enzyme-level mechanism, but not CSC depletion.	Limited: Direct molecular evidence; no CSC model.	[30]
Colorectal cancer cell lines	Prolonged native curcumin exposure	Both hypermethylation and hypomethylation occurred at selected CpG loci; no generalized global demethylation.	Shows locus- and cell-line-dependent epigenetic effects.	Limited for CSC-related inference; important qualifying evidence: The findings caution against calling curcumin a universal demethylating agent.	[37]
Acute myeloid leukemia cells, primary blasts, xenograft	Native curcumin	DNMT1 decreased through NF-κB/p65 and Sp1 regulation; p15INK4B was hypomethylated and re-expressed.	Cell-cycle arrest, apoptosis, and reduced tumor growth.	Limited: Direct curcumin–DNMT evidence; purified CSCs were not tested.	[42]
Breast cancer cells	Native curcumin	DNMT1 expression/activity decreased and RASSF1A was partially reactivated.	Restoration of a methylation-silenced tumor-suppressor program.	Limited: Direct molecular evidence; no causal CSC assay.	[43]
Cancer-cell model	Native curcumin	Demethylation of the p21Cip1 promoter region and enhanced KLF4 recruitment were reported.	Increased p21Cip1 expression.	Limited: Locus-specific evidence; not CSC-resolved.	[44]
Breast cancer cell lines	Native curcumin	BRCA1 re-expression was associated with altered promoter methylation.	Tumor-suppressor reactivation.	Limited: Model-specific; no CSC depletion or rescue study.	[45]
Breast cancer cells	Natural compounds, including curcumin	DNMT1, DNMT3A, and DNMT3B expression decreased, but the examined promoter methylation panel did not change.	Demonstrates that lower DNMT abundance does not automatically prove demethylation.	Very limited/qualifying: Reduced DNMT expression did not correspond to altered methylation in the examined promoter panel.	[46]
HepG2 and Huh7 hepatocellular carcinoma cells	Dendrosomal nanocurcumin	DNMT1/3A/3B decreased and miR-34 family expression increased.	Links formulation-dependent DNMT and miRNA regulation.	Limited: Nanoformulation; bulk cells; not direct CSC evidence.	[47]
Non-small-cell lung cancer cells	Native curcumin	DNMT1/3A/3B decreased; miR-142-5p increased; ULK1-associated autophagy was inhibited.	Increased crizotinib sensitivity.	Limited: Epigenetic sensitization in bulk cells, not CSC-specific.	[48]
Gastric cancer cells	Native curcumin	Demethylation was linked to a DNA-damage-response pathway involving p53, p21/GADD45A, E2F, and DNMT1.	Cell-cycle and transcriptional effects.	Limited: Mechanistically suggestive; no isolated CSC population.	[49]
Raji B-cell lymphoma cells	Native curcumin	HDAC1, HDAC3, and HDAC8 decreased; acetylated histone H4 increased.	Reduced proliferation and increased apoptosis.	Limited: Selected HDAC isoforms only; no CSC assay.	[52]
Medulloblastoma models	Native curcumin	Total HDAC activity and HDAC4 decreased; tubulin acetylation increased.	G2/M arrest, apoptosis, and reduced in-vivo tumor growth.	Limited: Direct HDAC-related evidence; not CSC-resolved.	[53]
Cell-free/chromatin systems	Native curcumin	p300/CREB-binding protein acetyltransferase activity was inhibited.	Critical evidence for context-dependent acetylation regulation.	Limited: Critical evidence for context-dependent acetylation regulation.	[54]
MDA-MB-435 cancer cells	Native curcumin	EZH2 expression decreased through mitogen-activated protein kinase signaling.	Growth-regulatory effect in a defined cell model.	Limited: Direct EZH2 evidence; no CSC population.	[31]
Myelodysplastic syndrome cell lines and xenografts	Native curcumin	EZH2 and H3K27me3 decreased and nuclear localization was altered.	Cell-cycle arrest, apoptosis, and reduced tumor growth.	Limited: Direct chromatin evidence; not direct CSC causality.	[58]
Lung cancer cells	Native curcumin	EZH2 transcription/mRNA stability decreased, partly with miR-101 and let-7c induction; NOTCH1 signaling was attenuated.	Reduced oncogenic signaling.	Limited: Mechanistic evidence in bulk cells; CSC link remains inferred.	[59]
Colorectal cancer cells	Native curcumin	BMI1 protein expression decreased.	Potential weakening of a Polycomb/stemness regulator.	Limited: No purified CSCs or BMI1 rescue experiment.	[61]

Evidence ratings were assigned according to the claim-level framework described in Section 1.1. Ratings apply to the specific CSC-related mechanistic claim, not to the overall quality of the cited study. No evaluated pathway met the criteria for Strong evidence. Abbreviations: CSC, cancer stem cell; DNMT, DNA methyltransferase; HDAC, histone deacetylase; H3K27me3, trimethylation of histone H3 lysine 27.

**Table 2:** Curcumin-regulated non-coding RNA axes and their relevance to CSC-associated phenotypes.

ncRNA	What the Curcumin Study Directly Showed	CSC/Plasticity Relevance	Claim That Must Be Avoided	Evidence Rating and Rationale	Refs.
miR-34a/b/c	Curcumin induced the miR-34 family through a ROS–KEAP1–NRF2 pathway in colorectal cancer cells; genetic deletion attenuated several curcumin responses.	CD44 and Notch-related CSC functions are supported by separate prostate and colon CSC studies.	Do not state that curcumin-induced miR-34a has already eradicated prospectively isolated CSCs.	Moderate for the direct curcumin–miR-34 mechanism; Limited for the integrated CSC claim: Direct curcumin mechanism + independent direct CSC evidence; integrated causal chain unproven.	[62,63,64]
miR-200c	Curcumin increased miR-200c and reduced EMT, migration, and invasion; EPM5 was the experimentally validated mediator in one study. Curcumin also increased EMT-suppressive miRNAs in 5-FU-resistant colorectal cancer.	The miR-200 family broadly regulates epithelial identity through ZEB1/ZEB2 in independent work.	Do not present therapeutic resensitization of isolated CSCs as established.	Moderate for the EMT-related mechanism; Limited for CSC depletion: Good curcumin–EMT evidence; CSC depletion remains indirect.	[65,66,67]
miR-21	Curcumin suppressed miR-21 and restored PDCD4 or PTEN-associated signaling in colorectal, breast, and gastric cancer cells.	These pathways influence survival, invasion, EMT, and treatment response	Do not present therapeutic resensitization of isolated CSCs as established.	Moderate for the direct molecular mechanism; Limited for CSC relevance: Direct curcumin–miR-21 evidence in bulk cancer cells.	[68,69,70]
miR-221/222	[71] used difluorinated curcumin (CDF), not native curcumin. [72] used a chitosan–cyclodextrin nanoformulation and showed cell-line-dependent responses.	miR-221/222 can regulate proliferation and tumor-suppressor pathways.	Do not generalize this as universal suppression by unformulated curcumin.	Very limited/hypothesis-generating for a native-curcumin CSC claim: Conditional evidence dependent on analogue, formulation, and cell type.	[71,72]
H19	Curcumin decreased H19 in gastric cancer cells; H19 overexpression partially reversed growth inhibition. Curcumin also attenuated H19-associated EMT in tamoxifen-resistant breast cancer cells.	H19 is associated with EMT and resistance-related phenotypes.	CSC maintenance was not directly measured.	Moderate for the functional curcumin–H19 relationship; Limited for CSC relevance: CSC relevance is inferential.	[73,74]
HOTAIR	Renal carcinoma cell migration varied with HOTAIR expression and curcumin exposure.	HOTAIR can influence migration and plasticity.	The study does not justify the statement that curcumin directly downregulates HOTAIR.	Very limited/hypothesis-generating: Association/modifier evidence; direct expression suppression not established.	[75]
MALAT1	MALAT1 silencing increased the inhibitory effects of curcumin on SW480 cell viability, migration, and invasion.	MALAT1 may modify responsiveness to curcumin.	Do not state that curcumin itself downregulates MALAT1.	Very limited/hypothesis-generating: Combination/cooperation evidence, not direct regulation.	[76]
circHN1	Curcumin reduced circHN1; restoration of circHN1 partially reversed curcumin effects through the circHN1–miR-302a-3p–PIK3R3 axis.	Proliferation, migration, invasion, EMT, and xenograft growth were affected.	No direct CSC self-renewal or limiting-dilution assay was performed.	Moderate for the rescued molecular mechanism in a non-CSC model; Limited for CSC relevance: Relatively strong mechanistic rescue evidence in a non-CSC model.	[77]

Evidence ratings were assigned according to the claim-level framework described in Section 1.1. Ratings apply to the specific CSC-related mechanistic claim, not to the overall quality of the cited study. No evaluated pathway met the criteria for Strong evidence. Abbreviations: 5-FU, 5-fluorouracil; CDF, difluorinated curcumin; CSC, cancer stem cell; EMT, epithelial–mesenchymal transition; miRNA, microRNA.

**Table 3:** Proposed experimental framework for validating curcumin-induced epigenetic destabilization of CSC plasticity.

Claim to Validate	Preferred Model	Essential Measurement	Required Causal Test	Minimum Success Criterion
CSC identity	Prospectively isolated CSC-enriched and matched non-CSC populations; patient-derived organoids where possible	Serial tumorsphere/organoid formation, differentiation, clonogenic regrowth, and validated marker panels	Depletion and rescue of the proposed regulator; marker changes alone are insufficient	Durable loss of self-renewal across serial passages
DNA methylation	CSC-resolved populations before and after curcumin exposure	Whole-genome or targeted bisulfite analysis with matched transcriptomics	DNMT rescue, CRISPR epigenome editing, or locus-specific reversal	A defined methylation change must be necessary for reduced self-renewal/tumor initiation
Histone/Polycomb regulation	CSC-enriched cells and organoids	CUT&RUN/ChIP-seq for relevant marks, ATAC-seq, and expression profiling	Isoform-specific HDAC, EZH2, or BMI1 rescue/depletion	Chromatin change linked causally to differentiation or loss of tumor-repopulating capacity
Non-coding RNA	CSC-resolved and bulk-cell comparisons	Small-RNA sequencing and long-RNA profiling with direct target validation	miRNA mimic/inhibitor or lncRNA/circRNA rescue	Reversal of curcumin phenotype by restoring the specific ncRNA axis
Plasticity and EMT	Time-resolved lineage or state-transition models	Epithelial/mesenchymal markers, live-cell lineage tracing, invasion, and reversible state transitions	Withdrawal and re-challenge experiments; pathway rescue	Reduced capacity to enter or recover a drug-tolerant stem-like state
Therapeutic sensitization	Clinically relevant resistant models and matched parental controls	Clonogenic survival, serial regrowth, apoptosis, and pharmacological interaction analysis	Mechanism-specific rescue and schedule controls	Durable sensitization at achievable exposure rather than short-term viability loss
In-vivo tumor initiation and relapse	Orthotopic or patient-derived xenografts with post-treatment follow-up	Limiting-dilution tumor initiation, serial transplantation, metastatic burden, and recurrence latency	Re-expression/rescue of the proposed epigenetic regulator	Reduced CSC frequency and delayed relapse after treatment cessation
Pharmacology/formulation	Native curcumin and formulation-matched controls	Free curcumin, metabolites, stability, release, tumor exposure, and toxicity	Empty-carrier and exposure-matched controls	Mechanistic effects reproduced at measured, pharmacologically credible concentrations

## Data Availability

No new data were created or analyzed in this study. Data sharing is not applicable to this article.

## Abbreviations

The following abbreviations are used in this manuscript:

CSCs	Cancer stem cells
ncRNA	Non-coding RNA
DNMT	DNA methyltransferase
HDAC	Histone deacetylase
EZH2	Enhancer of zeste homolog 2
H3K27me3	Histone H3 lysine 27 trimethylation
miRNA	MicroRNA
lncRNA	Long non-coding RNA
circRNA	Circular RNA
EMT	Epithelial–mesenchymal transition
BMI1	BMI1 proto-oncogene, Polycomb ring finger
HOTAIR	HOX transcript antisense intergenic RNA
MALAT1	Metastasis-associated lung adenocarcinoma transcript 1
CpG	Cytosine–phosphate–guanine
NF-κB	Nuclear factor kappa B
Sp1	Specificity protein 1
G1	Gap 1 phase
PI3K/Akt	Phosphoinositide 3-kinase/protein kinase B
G2/M	Gap 2/mitosis transition
CBP	CREB-binding protein
ERK	Extracellular signal-regulated kinase
MAPK	Mitogen-activated protein kinase
IL-6R	Interleukin-6 receptor
PRC1	Polycomb repressive complex 1
ROS	Reactive oxygen species
KEAP1	Kelch-like ECH-associated protein 1
NRF2	Nuclear factor erythroid 2-related factor 2
AP-1	Activator protein 1
CDF	Difluorinated curcumin
Hsp90	Heat shock protein 90
